# Synthesis, Drug Release, and Antibacterial Properties of Novel Dendritic CHX-SrCl_2_ and CHX-ZnCl_2_ Particles

**DOI:** 10.3390/pharmaceutics13111799

**Published:** 2021-10-27

**Authors:** Rui Sun, Jiaxin Zhang, Robert A. Whiley, Gleb B. Sukhorukov, Michael J. Cattell

**Affiliations:** 1 Centre for Oral Bioengineering, Bart’s and the London, School of Medicine and Dentistry, Queen Mary University of London, Turner Street, London E1 2AD, UK; rui.sun@qmul.ac.uk; 2 School of Engineering and Materials Science, Queen Mary University of London, Mile End Road, London E1 4NS, UK; jiaxin.zhang@qmul.ac.uk; 3 Centre for Oral Immunobiology and Regenerative Medicine, Bart’s and the London, School of Medicine and Dentistry, Queen Mary University of London, Blizzard Building, 4 Newark Street, London E1 2AT, UK; r.a.whiley@qmul.ac.uk

**Keywords:** chlorhexidine, drug delivery, controlled release, particulate delivery systems, antibacterial

## Abstract

This work demonstrated for the first time the synthesis of novel chlorhexidine particles containing strontium and zinc, to provide an effective, affordable, and safe intervention in the treatment of recurrent infections found in Medicine and Dentistry. The CHX-SrCl_2_ and CHX-ZnCl_2_ particles were synthesized by co-precipitation of chlorhexidine diacetate (CHXD) and zinc chloride or strontium chloride, where particle size was manipulated by controlling processing time and temperature. The CHX-ZnCl_2_ and CHX-SrCl_2_ particles were characterized using SEM, FTIR, and XRD. UV-Vis using artificial saliva (pH 4 and pH 7) was used to measure the drug release and ICP-OES ion release. The antibacterial properties were examined against *P. gingivalis*, *A. actinomycetemcomitans*, and *F. nucleatum subsp. Polymorphum*, and cytotoxicity was evaluated using mouse fibroblast L929 cells. The novel particles were as safe as commercial CHXD, with antibacterial activity against a range of oral pathogens. UV-Vis results run in artificial saliva (pH 4 and pH 7) indicated a higher release rate in acidic rather than neutral conditions. The CHX-ZnCl_2_ particles provided the functionality of a smart Zinc and CHX release, with respect to environmental pH, allowing responsive antibacterial applications in the field of medicine and dentistry.

## 1. Introduction

Antiseptics are widely applied for preventing and reducing wound and surgical infections, which continue to be a major problem in health care [1]. This can be particularly difficult to manage in developing countries due to lack of resources, high costs, and special storage conditions of antibacterial agents [2]. Lack of suitable antibacterial agents can however lead to high infection rates and even death [3], due to ventilator associated pneumonia and sepsis [4]. An effective, affordable, and safe intervention for preventing infections and improving public health is therefore in high demand.

Chlorhexidine (CHX) is an antibacterial agent [5,6,7] widely applied in medicine and dentistry [8,9]. It has been used for daily bathing of critically ill patients in intensive care units [10] or as a neonatal wipe for cord care [11]. In the dental field, it is used to control dental plaque [12] and reduce surgical/recurrent infections (e.g., periodontal disease and endodontic disease) [13,14,15,16]. As a broad-spectrum antibacterial agent, CHX provides both bactericidal and bacteriostatic effects by producing non-specific binding to the negatively charged membrane phospholipids of the bacteria. This leads to alteration in bacterial osmotic equilibrium. When the CHX concentration is increased the cytoplasm contents precipitate, causing cell death [17,18].

Many systems have been proposed for chlorhexidine delivery, where the direct addition of chlorhexidine gluconate solution (4%) [19] is used for burns and wounds, or 0.12–0.3% CHX in a mouthwash as common methods of reducing bacterial load [20,21]. Generally, the incorporation of CHX gluconate into loading systems involves gels and varnishes. Biodegradable chips or incorporation into polymers are also feasible methods [22,23]. The low drug loading capacity, burst drug release, and strong binding formed between the drug and the polymeric matrix, however, limits its antibacterial efficacy [24,25]. To achieve effective chlorhexidine delivery, modification of the chlorhexidine formulation is one of the promising and feasible strategies. Previous studies suggested the co-precipitation of CHXD with CaCl_2_ to produce CHX-CaCl_2_ spheres, which demonstrated sustained release behavior and high CHX content (95%) [26]. It is also possible to substitute Ca^2+^ ions within the structure with other ions, to bring additional therapeutic benefits. Good candidates for this particle substitution are ions such as Zn^2+^ and Sr^2+^ to optimize antimicrobial activity [27,28,29].

The presence of strontium (Sr) would be beneficial to perform the dual action of bone stimulation and bone resorption suppression [30,31]. Numerous studies demonstrate strontium is able to promote pre-osteoblast proliferation, osteoblast differentiation, bone matrix mineralization, and stimulate type I collagen protein levels [32], while inhibiting osteoclast differentiation [33]. Therefore, strontium inclusion would be particularly useful in the treatment of implant and surgical associated infections (peri-implantitis) found in dentistry. Strontium has also been shown to significantly enhance the remineralizing effect of fluoride ion (F^−^) [34] and to help remineralize dentine in vitro [35].

Zinc (Zn) has various positive characteristics, such as angiogenic, osteogenic, and antimicrobial properties [36]. Zn^2+^ is attracted to the negatively charged microbe cell membrane, penetrates it, and reacts with sulfhydryl within it. Thus, the activity of synthetase in the microbe is damaged and the cells lose the ability of mitosis, which leads to death of the microbe [37]. In practice, owing to its bacteriostatic properties, zinc is incorporated in dental filling materials, mouth rinses, and toothpastes [38]. The release rate of Zn increases dramatically under acidic conditions in Zn containing silicate glasses, enabling Zn release during bacterial infections at low pH value (pH = 4.5) [39]. This particular property would also be very attractive in the development of a smart release antiseptic.

Therefore, the aim of the work is to use low-cost reagents and energy-saving synthesis to develop a novel drug delivery particle that can pH responsively release antibacterial agents of CHX and metal ions to take advantage of their therapeutic functions, thus providing a safe and effective antimicrobial environment for dental and medical applications.

## 2. Materials and Methods

### 2.1. Materials

Chlorhexidine diacetate (C6143, Lot: 4G013891), strontium chloride hexahydrate (204463, Lot MKC131848V); zinc chloride (229997; Lot MKCC2307), KH_2_PO_4_ (P3786, Lot #BCBC2041), Hepes (lot SLBW8459), KCL (Lot BCBZ4557), and phosphate-buffered saline (PBS, Lot 1762519) were all purchased from Sigma-Aldrich (Gillingham, Dorset, UK). All the solutions were prepared using deionized (DI) water (Milli-Q, Millipore Co., Bedford, MA, USA) with an 18.2 MΩ·cm resistance.

### 2.2. Chlorhexidine Particle Synthesis

The chlorhexidine (CHX) particles were synthesized by coprecipitation of 15 mg/mL chlorhexidine diacetate (CHXD) with 0.33 M of SrCl_2_ or ZnCl_2_, mixed at 1:1 by volume at room temperature. The mixtures were left for 1 min and then centrifuged at 2400 rpm for 1 min (Eppendorf centrifuge 5417C, Germany). After wash with 1 mL deionized water, the precipitates were placed into liquid nitrogen for 30 min, and then transferred to a freeze dryer (ScanVac Cool Safe Freeze Drying, Denmark) at −107 °C, 0.009 mBar for 24 h. Both strontium (CHX-SrCl_2_) and zinc containing particles (CHX-ZnCl_2_) were synthesized following the same procedures. The synthesized particles were then stored at 4 °C and wrapped in aluminum foil paper to exclude the light, ready for further characterization.

To evaluate the influence of temperature on CHX-SrCl_2_ and CHX-ZnCl_2_ particle formation, both CHXD and SrCl_2_/ZnCl_2_ solutions were kept in a temperature-controlled water bath or ice bath (IKA RET basic C, UK) at selected temperature points of 0, 5, 10, 15, 20, and 25 °C. The coprecipitation process was then carried out at these selected temperatures using the same procedures and storage as previously.

To demonstrate the influence of the reaction times on the formation of both particles, 20 μL CHXD solution (15 mg/mL) was added onto glass slides and left for one minute, 20 μL SrCl_2_ or ZnCl_2_ (0.33 M) was further added and reacted for 15 s, 30 s, 45 s, and 60 s. Then the excess liquid was carefully removed with fuzz-free lab wipes. Statistical differences between particle diameter for each test group (reaction time or temperature) were determined using a one-way Anova test (*p* < 0.05, SPSS Inc., Chicago, IL, USA).

### 2.3. UV-Vis Spectrometer

For CHX concentration determination, a series of CHXD standard solutions (1, 3, 5, 10, 20, 30, and 50 ppm) were prepared and measured using UV–Vis spectrometry (Lambda 35, Perkin Elmer, Waltham, MA, USA). The measured data were used to obtain a linear calibration curve between the absorbance intensity (at 254 nm) and the CHX concentration.

The release kinetics of the particles were tested by weighing 0.01 g of CHX-SrCl_2_ or CHX-ZnCl_2_ particles into 2 mL centrifuge tubes containing 2 mL phosphate buffered saline. At specified time points (Table S1), the samples were centrifuged, and the supernatant was collected for UV−Vis spectrometry measurements. Fresh PBS (2 mL) was then replaced, and the procedure repeated at the next time points. Cumulative release curves were formed by further calculation of the CHX release at each time point. The yield of CHX was calculated using the overall amount of CHX in the original solutions subtracted from the amount of CHX left in the synthesis supernatant and washing solutions.

The release behavior of the CHX-ZnCl_2_ particles was also tested in artificial saliva (AS) of pH 4 and pH 7. First, 0.04 g of CHX-ZnCl_2_ particles were placed into 15 mL centrifuge tubes (Eppendorf, Germany), containing 8 mL AS (pH = 4 or pH = 7) and the solutions were replaced as per the previous timetable. At each time point samples were centrifuged, and the supernatants were measured using UV-Vis spectrometry to gather the CHX concentration and to calculate the cumulative CHX release.

### 2.4. Inductively Coupled Plasma-Optical Emission Spectroscopy

Elemental calibration standards were prepared from pure element standards (1000 ppm standards in deionized water, VWR), which were diluted in deionized water to produce 0, 1, 5, 10, 50, and 100 ppm concentrations of each ion. The zinc content of the calibration standards was measured using inductively coupled plasma-optical emission spectroscopy (ICP-OES; Varian Vista-PRO, Yarnton, UK).

ICP-OSE was employed for measuring the zinc release curve of CHX-ZnCl_2_ particles in AS of pH = 4 and pH = 7, according to the time points in Table S1 (up to 7 h). At each time point, samples were centrifuged, and the supernatants were collected and replaced with fresh AS. All the experiments were repeated in triplicate.

### 2.5. Structural Analysis of the CHX-SrCl_2_ and CHX-ZnCl_2_ Particles

The morphology of CHX-SrCl_2_, CHX-ZnCl_2_ particles, and prepared tissue samples were evaluated using scanning electron microscopy (SEM, FEI inspect-F, Hillsboro, USA), after gold coating using a sputter coating system (SC 7620, Quorum, Laughton, UK), for 45 s at 20 mA. Samples were analyzed at 10 kV, with a spot size of 3.0. SEM photomicrographs were taken to assess the different morphology, and size distribution of the CHX-SrCl_2_ and CHX-ZnCl_2_ particles at selected temperatures and different reaction times.

Elemental analysis of the CHX-SrCl_2_ and CHX-ZnCl_2_ particles was conducted using Energy Dispersive Spectroscopy (EDS, FEI Inspect F, NanoPort, Eindhoven, The Netherlands). X-ray diffraction (XRD) was employed using an X’Pert Pro X-ray Diffractometer (Panalytical, Almelo, The Netherlands) to characterize the structural information of CHXD and novel CHX particles. Fourier Transform Infrared Spectroscopy (FTIR, Bruker, Billerica, MA) was further applied to analyze the chemical structures of the CHX-SrCl_2_ and CHX-ZnCl_2_ particles.

### 2.6. Antimicrobial Assay

*Porphyromonas gingivalis* (strain-381), *Fusobacterium nucleatum subspecies nucleatum* (strain-ATCC10953), and *Aggregatibacter actinomycetemcomitans* (strain-Y4) were used to test the antibacterial activity of CHXD, CHX-SrCl_2_, and CHX-ZnCl_2_ particles. Bacteria were grown on blood agar plates (Blood Agar Base No.2; Oxford, UK) and 5% defibrinated horse blood (TCS, UK) in an anaerobic atmosphere (10% H_2_, 10% CO_2_, and 80% N_2_), at 37 °C for 48 h. The resulting colonies were inoculated and suspended into 20 mL brain heart infusion broth (BHI) (CM1135; Oxford, UK), supplemented with 0.5 μg/mL Vitamin K and 0.5 μg/mL hemin. The bacterial culture was then grown for 24 h anaerobically. Bacterial numbers in the BHI broth were determined and standardized by serial dilution and measured for colony forming units (CFUs) on blood agar plates. After overnight incubation in an anaerobic environment, the bacterial suspensions were diluted (1:20) in pure BHI, to achieve an optical density of 0.1 for *Porphyromonas gingivalis* and *Aggregatibacter actinomycetemcomitans*, and 0.2 for *Fusobacterium nucleatum* at 600 nm (OD_600_). This gave approximately 6.36 × 10^7^ colony-forming units (CFU) per ml, to standardize the bacterial inoculum used in these experiments.

The MIC (minimum inhibitory concentration) is the lowest or minimum antimicrobial concentration that inhibits visible microbial growth in artificial media after a fixed incubation time. This was determined by placing a known quantity of bacteria in 96 well plates, and then adding a series of dilutions ranging from 0.0000625–0.004% of CHXD powder, CHX-SrCl_2_, or CHX-ZnCl_2_ particles in sterile deionized water. The test wells were (a) bacterial suspensions with 225 μL BHI and 25 μL of CHXD, CHX-SrCl_2_, or CHX-ZnCl_2_ particle dilutions (0.0000625–0.004%); Controls with: (b) 225 μL BHI and 25 μL sterile water; (c) bacterial suspension with 225 μL BHI; (d) bacterial suspension with 225 μL BHI and 25 μL of sterile water. All experiments had six duplicate wells and three independent experiments. The plates were incubated for 24 h in an anaerobic incubator and OD was measured at 595 nm (OD_595 nm_), to calculate the bacterial growth. After incubation the MBC was confirmed by transferring the microliter well contents to microcentrifuge tubes, then centrifuging the bacterial suspension solution (5 min). The bacteria were washed to remove any remaining CHX-SrCl_2_, CHX-ZnCl_2_, particles or CHXD, then re-suspended on blood agar plates. After 24–72 h incubation, bactericidal activity was confirmed by observation of any bacterial colonies on the culture plate. When 99.9% of the bacterial population was killed at the concentration of the CHXD or novel CHX particles, it was termed the MBC (minimum bactericidal concentration). This was done by observing pre- and post-incubated agar plates for the presence or absence of bacteria.

### 2.7. Cytotoxicity Assay

Cytotoxicity of the CHX particles was evaluated with a standard 3-(4,5 dimethylthiazol-2-yl)-2,5-diphenyltetrazolium bromide (MTT) assay with the L929 cell line (ECACC, 85011425). The MTT activity reflects mitochondrial activity, which can indicate viable cells. The cell viability of CHX-SrCl_2_, CHX-ZnCl_2_, and CHXD particles were tested using three independent experiments each, with six replicate wells for all antimicrobial agent concentrations tested. Cells were cultured in Dulbecco’s modified eagle’s medium (DMEM, Lonza, Switzerland), supplemented with 10% fetal bovine serum (FBS), 100 μg/mL penicillin, and 100 μg/mL streptomycin. This was carried out in a humidified incubator atmosphere (5% CO_2_ at 37 °C), then seeded in 96-well microliter plates at 10,000 cells per well. The cell was incubated overnight, after that the medium was removed, and cells were washed by PBS twice. CHX-SrCl_2_, CHX-ZnCl_2_, and CHXD particles were separately dissolved in sterilized DI water, then diluted in cell culture media to get the concentrations from 0.0000625 to 0.004%. The treatment solutions were used to treat cells for 24 and 48 h. After this, treatments were removed, and 50 μL of 5 mg/mL tetrazolium salt MTT was added into each well and then incubated at 37 °C for 4 h. The medium was next removed, and MTT was solubilized in 100 μL isopropanol in each well. The absorbance of the solution was measured with a plate reader at 570 nm.

For each test, results were expressed as mean (SD)%. The original optical density of the examination culture was calculated as a percentage of the control medium optical density. It is assumed that the absorption value of the control group represents 100% viability. Statistical differences between the Zn or Sr containing particles and CHXD within concentration groups were determined using independent *t*-tests (SPSS Inc., Chicago, IL, USA).

To demonstrate the influence of novel CHX particle synthesis on tissue adherence, three tissue sections (10 mm length × 6 mm depth) were removed from the lingual wall of a lower jaw of a pig’s head, at 2 mm from the gingival margins, using a scalpel (Swann Morton, Sheffield UK). The tissue sections were mounted onto metal SEM stubs using copper tape. Human saliva (20 mL) was collected from underneath the tongue and placed in a 30 mL universal container (Star lab, UK) and stored at 37 °C in an incubator. A micro-brush (Stewmac #3101, UK) was dipped into a dappens pot containing 1 mL of human saliva and it was then applied to the surface of all the tissue mounted samples.

One tissue section was used as a control group without any particle coating. The other two tissue sections were coated with CHX-ZnCl_2_ and CHX-SrCl_2_ particles respectively. CHX particles were synthesized onto the surface of the moisturized tissue sections and also on a separate SEM stub (Figure S7), by the co-precipitation of 10μL CHXD solution (15 mg/mL) and 10μL of 0.33 mol/L of either ZnCl_2_ or SrCl_2_ solutions. Then, the solution was then removed by syringing with 2 mL human saliva drop by drop within 4 min.

## 3. Results and Discussion

### 3.1. Synthesis and Characterization of CHX-SrCl_2_ and CHX-ZnCl_2_ Particles

CHXD and chlorhexidine di-gluconate are the common components of antibacterial drugs that have been extensively applied in the field of medicine and dentistry [40,41]. The authors previously proposed a novel method for preparation of spherical CHX-CaCl_2_ particles, using CHXD to coordinate with CaCl_2_ [26]. The incorporated calcium ions, however, may hold less significant antibacterial and therapeutic functions. In the present work, a novel method of producing functionalized spherical CHX particles is demonstrated by substituting zinc and strontium ions into the particle structure to enhance antibacterial effects. Strontium and zinc ions are both divalent metal ions with ionic radii (Sr = 1.16 Å, Zn = 0.74 Å) close to calcium (0.94 Å) [42,43] facilitating this substitution. The substitution of calcium by strontium and zinc has become a feasible route, especially in the application of bioactive glasses [44,45].

**Figure 1:**
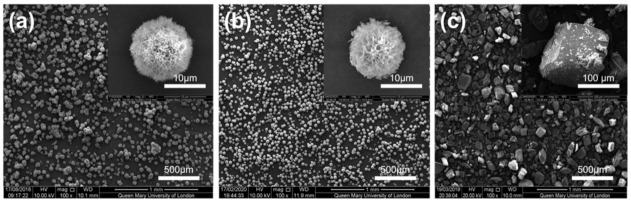
SEM images of (**a**) CHX-SrCl_2_ particles; (**b**) CHX-ZnCl_2_ particles; (**c**) CHX diacetate particles.

The CHX-SrCl_2_ and CHX-ZnCl_2_ particles were evaluated using scanning electron microscopy (SEM) with the results shown in Figure 1. Generally, both CHX-SrCl_2_ and CHX-ZnCl_2_ particles demonstrated unique spherical morphologies of a porous and interconnected dendritic structure, grown from a nucleation site central to the sphere (Figure 1a,b). This was in contrast to the angular shaped solid morphology of original CHXD platelet (Figure 1c). The mean (SD) diameters of the prepared CHX particles (synthesized at 25 °C) were: 17.5 (4.39) μm (CHX-SrCl_2_) and 14.2 (4.71) μm (CHX-ZnCl_2_), which were significantly smaller than original CHXD crystals of 80 (30) μm.

**Figure 2:**
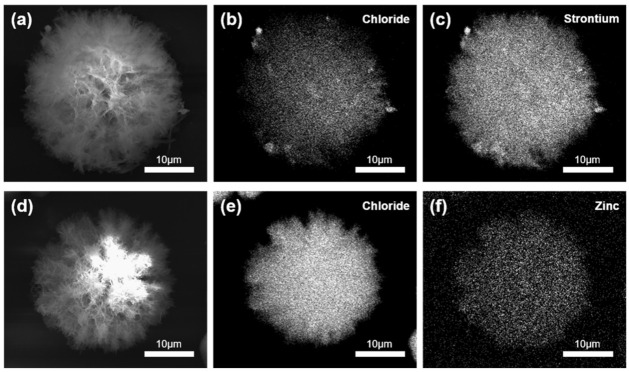
(**a**) SEM photomicrograph of CHX-SrCl_2_ particle; (**b**) EDS mapping of chloride in CHX-SrCl_2_ particle; (**c**) EDS mapping of strontium; (**d**) SEM photomicrograph of CHX-ZnCl_2_ particle; (**e**) EDS mapping of chloride in CHX-ZnCl_2_ particle; (**f**) EDS mapping of zinc.

The EDS results of the CHX-SrCl_2_ particles (Figure 2a–c) indicated an even distribution of strontium and chloride throughout the particle and associated with its structure. Similarly, Figure 2d–f demonstrated a homogeneous distribution of zinc and chloride ions in the CHX-ZnCl_2_ particles. Quantitative elemental analysis indicated that the weight percentage of divalent ions incorporated in the CHX particles was 3.95 wt% and 7.66 wt% for strontium and zinc, respectively (Figures S1 and S2, Tables S2 and S3). Additionally, it is noteworthy to mention that the CHX-SrCl_2_ and CHX-ZnCl_2_ particles were washed with DI water to remove unreacted elements and any physically absorbed divalent metal ions. The zinc and strontium ions were therefore confirmed evenly incorporated into particle structures.

**Figure 3:**
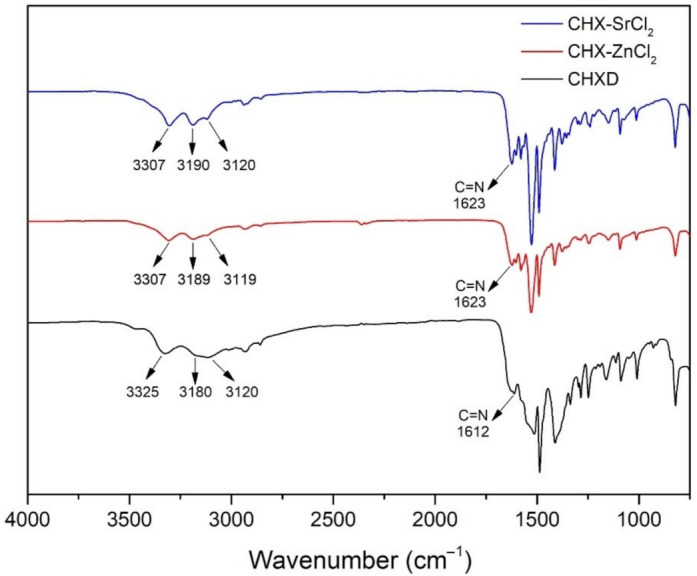
FTIR results of CHX-SrCl_2_, CHX-ZnCl_2_, and CHXD particles.

The FTIR results of the CHX-SrCl_2_, CHX-ZnCl_2_, and CHXD particles are shown in Figure 3. In the infrared spectrum of the CHXD, there are three absorption peaks at 3325 cm^−1^, 3120 cm^−1^, and 3180 cm^−1^, which may be attributed to the stretching vibrations N–H of the groups (Alkyl)_2_ NH, Alkyl-NH-Aryl, and to the group =NH, respectively [46]. A typical band at 1612 cm^−1^ can also be assigned to the stretching vibration of the imine group C=N [47,48]. The IR spectrum of the CHX-SrCl_2_ and CHX-ZnCl_2_ particles for the band of the imine group displays a positive shift from 1612 cm^−1^ to 1623 cm^−1^. In addition, after forming the novel CHX particles, the stretching vibrations N–H of the groups (Alkyl)_2_ NH and the group =NH showed significant shifts as well. Based on the comparisons between the infrared spectrums of the CHX-SrCl_2_, CHX-ZnCl_2_ particles, and CHXD, it is inferred that the electron density was affected and changed the position of –C=NH in favor of the addition of metal ions. Coordination might therefore be formed between the biguanides of the novel CHX particles and the divalent metal ions through the formation of four metal–N bonds with two bidentate ligands in the square-planar structure [49,50]. A similar phenomenon of chelated chlorhexidine complexes has been previously found and the mechanisms discussed [28,29,51]. EDS analysis of CHX-SrCl_2_ and CHX-ZnCl_2_ particles also confirms the structural presence and homogeneous distribution of the metal cations and chloride. It can be assumed that the coordination of chlorhexidine to the divalent metal (Zn, Sr) ions happened during particle formation. Consequently, both the negatively charged chloride ions and the counterpart divalent metal ions are responsible for the formation of novel chlorhexidine particles. EDS revealed a higher ratio (25.4–28.3 at%) of Cl^−^ ions compared to cations distributed within the particles (Tables S2 and S3). The additional chloride ions may reduce the solubility of the chlorhexidine as well as influence the rate of structural formation, whilst the metal ions may contribute to the production of the compactness of spherical microstructure as previously proposed [26].

**Figure 4:**
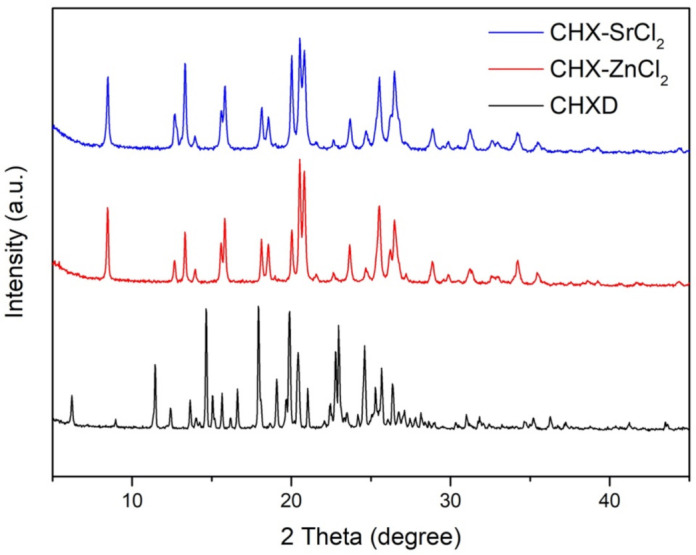
The X-ray diffraction results for CHX-SrCl_2_, CHX-ZnCl_2_, and CHXD particles.

The XRD plots of the CHX-SrCl_2_ and CHX-ZnCl_2_ particles indicated they had missing peaks at the 6.20, 11.40, 14.47, 16.18, 16.61, 19.09, and 36.27 degree 2 theta positions, which were present in the CHXD XRD plot. There were also slight deviations in the 2 theta positions, changes in intensity for similar peaks for the CHXD and the novel particles, and signs of peak broadening (Figure 4). Crystal lattice strain and crystal size can affect XRD peak broadening, the intensity of the peaks and shifts in 2 theta positions [52]. XRD peak broadening has been previously associated with smaller crystallite size and changes to the lattice parameters, due to zinc incorporation into hydroxyapatite structure [53]. The CHX-SrCl_2_ and CHX-ZnCl_2_ particles also displayed new peak positions (not in the CHXD plot) at 15.81, 15.85, 18.55, 20.02, 20.83, 21.56, 28.85, 28.88, 29.85, 29.88, 31.22, 35.43, and 35.46 degrees 2 theta. The CHX-SrCl_2_ and CHX-ZnCl_2_ particles displayed almost the same 2 theta positions, indicating the formation of a similar particle structure after the incorporation of divalent metal ions (Sr, Zn). It is clear the new particle complexes displayed structural differences with CHXD which were confirmed by differences in polymorph structure and crystallite size (Figure 1).

### 3.2. Influence of Reaction Time and Temperature on Particle Size

**Figure 5:**
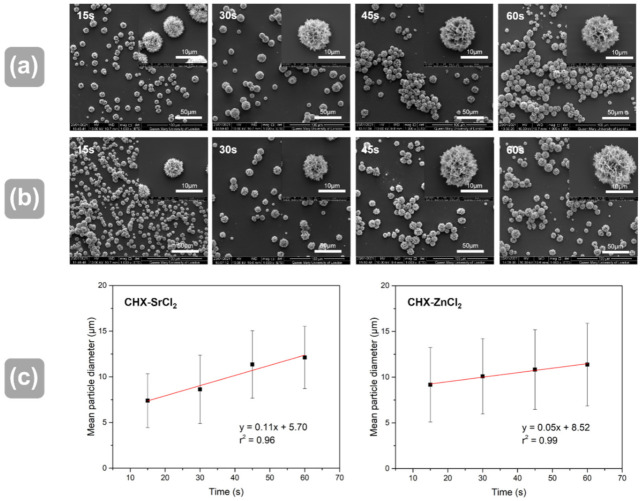
SEM images of (**a**) CHX-ZnCl_2_ particles; (**b**) CHX-SrCl_2_ particles at different reaction times; and (**c**) plots showing the correlation between the mean particle diameter and reaction time for both CHX-SrCl_2_ and CHX-ZnCl_2_ particles.

Reaction time and temperature are factors that can affect the crystallization processes. The crystallization reaction of the CHXD and SrCl_2_/ZnCl_2_ solutions appeared to be extremely rapid (turbidity in 0.05 secs, Figure S3), with a statistical increase in mean particle diameter (*p* < 0.05) with increased reaction time (Figure 5a–c). Both particles showed dendritic and spherical structures and statistical differences in mean particle diameter between all reaction time groups (*p* < 0.05). The CHX-SrCl_2_ and CHX-ZnCl_2_ particles were also produced at selected temperature points (0 °C to 25 °C), and the results are shown in Figure S4. There was a statistical increase in mean particle diameter with increase in synthesis temperature for both particles (Figure S4a–c). Both particles showed statistical differences in mean particle diameter between all selected temperature groups (*p* < 0.05). This is due to the essential role that temperature plays in the nucleation and crystal growth process [26]. Specifically, increased temperature will not only encourage the movement of ions, thereby speeding up the rate of crystal growth, but also decrease nucleation centers and increase the critical size of the nucleus, resulting in the formation of particles of larger size [54,55]. At lower temperatures, both the molecular movement and the critical nucleus size will be reduced, which may facilitate molecules binding and attaching impurities in their structure, forming a large number of smaller crystals [56]. This may partially explain the results in Figure S4a–c, as at 0 °C the novel particles exhibited the formation and agglomeration of smaller crystallites, whereas crystal growth increased in response to higher temperatures.

Crystallization of the current CHX-SrCl_2_ and CHX-ZnCl_2_ particles required a limited reaction time and energy input to produce spherical particles. This reactive crystallization may in part be due to the differing pH of the solutions used (pH 7.42 for CHXD-15 mg/mL, pH 5.03 for SrCl_2_ and pH 5.31 for ZnCl_2_), producing formation of a lower solubility solute with higher concentration and allowing crystallization [56]. These pH differences are significant since the pH scale is a logarithmic and therefore a 1 pH unit increase leads to tenfold increases in H^+^ concentration. Jiang et al. 2005 [57] indicated the particle size of SnO_2_ nanoparticles via colloids could similarly be controlled from 6 to 12 nm by varying pH value from 2 to 6. This co-precipitation process requires much higher nucleation and growth times/temperatures and mechanical agitation [58], to control particle size and distribution [56]. The present work provides a simple method to control particle size/surface area, so structure property particle relations can be tailored for applications, such as electro spinning, coating, or incorporation in microcontainers for drug delivery [59,60].

### 3.3. Antibacterial and Cytotoxicity Assay

For an antimicrobial agent to be efficacious in the treatment of a disease, it must be effective against the involved pathogens. The antimicrobial tests showed that the concentration of both CHX-SrCl_2_ and CHX-ZnCl_2_ particles required to inhibit (MIC) planktonic *P. gingivalis (strain-381)* and *A. actinomycetemcomitans (strain-Y4)* was 0.00025% at both 24 and 48-h time points, demonstrating their antibacterial efficacy in comparison to the CHXD (Figure S5). The concentration of CHX particles required to inhibit (MIC) planktonic *F. nucleatum subsp. polymorphum (strain- ATCC10953)* was 0.0005% at 24 and 48-h time points (Figure S5), again consistent with the efficacy of the commercial CHXD comparison antimicrobial.

The MBC results are shown in Table 1 (*P. gingivalis* and *A. actinomycetemcomitans*) and Table 2 (*F. nucleatum subsp. polymorphum*). The MIC and MBC of CHX-SrCl_2_ and CHX-ZnCl_2_ particles against *P. gingivalis*, *A. actinomycetemcomitans* and *F. nucleatum subsp. Polymorphum* were consistent with CHXD and in the range of 0.0000625–0.004%. The confirmation of MIC/MBC of CHX-SrCl_2_ and CHX-ZnCl_2_ particles against the bacteria tested provided an effective concentration range when conducting release experiments.

**Figure 6:**
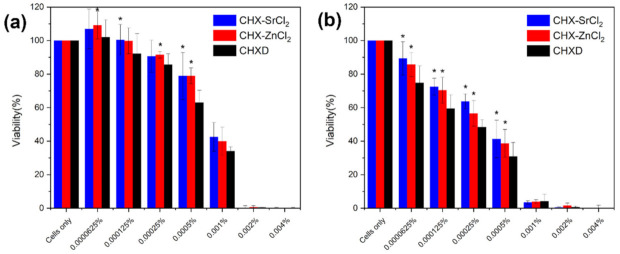
Effects of CHXD, CHX-SrCl_2_, and CHX-ZnCl_2_ on the relative viability of fibroblast (L929) cells at (**a**) 24 h and (**b**) 48 h. Values shown are mean (SD) for triplicate cultures. * *p* < 0.05 = significant difference within groups vs. CHXD.

The efficacy of chlorhexidine as an antibacterial drug is proven; however, tissue exposed to high CHX concentrations (>0.5–2%) [61] for long periods, may have adverse effects on the oral tissues [62,63]. The potential cytotoxic effects and safe concentration levels of the novel particles were, therefore, evaluated. There were significant differences (*p* < 0.05) between the novel particles and CHXD at 0.0005% at 24 h, and the concentrations from 0.0000625% to 0.0005% at 48 h (Figure 6a,b).

Application of the CHXD, CHX-SrCl_2_, and CHX-ZnCl_2_ particles reduced the viability of the L929 cells in a dose-dependent manner, but to different degrees (Figure 6). Relative cellular viability was reduced to approximately 40% when 0.001% of CHX-SrCl_2_ and CHX-ZnCl_2_ particles were used for 24 h, which was further decreased to around 2% after 48 h treatment.

Similar results were reported for human gingival fibroblast cells indicating decreased cell proliferation and division when exposed to 0.01%–0.02% CHX for 15 min [64]. When evaluating the cell structure of the fibroblast cells exposed to the current CHXD solutions (>0.01%), the cells’ shape became more rounded and less prolific (Figure S6). Characteristic fibroblast spindle shaped morphology was previously shown to lack filopodia and present a more oval or rounded shape at 0.002–0.04% CHX concentration, indicating a significant influence of CHX concentration on cellular viability and morphology [64,65,66].

Lower concentrations of CHX-SrCl_2_ and CHX-ZnCl_2_ particles ranging from 0.0000625% to 0.00025% showed approximately 90% and 60% cellular viability at 24 and 48 h, respectively. Although 0.0005% CHX-SrCl_2_ and CHX-ZnCl_2_ particles demonstrated above 80% cellular viability at 24 h, this was reduced to around 40% at 48 h. Similar viability was observed in CHXD treated cultures. Interestingly, a CHX concentration of 0.00025% caused no reported apoptosis and necrosis in L929 fibroblasts [65], and no necrosis at 0.125% CHX [65]. Current commercial products contain 0.12%–0.3% [20,21] CHX for mouthwashes and up to 4% for burns [67,68] which could significantly reduce cell viability. Furthermore, 0.05% CHX was reported as non-toxic to wound healing and granulation tissue [19], with no suggested bioaccumulation after repeated exposure at higher CHX levels [69]. Therefore, the current CHX-SrCl_2_, CHX-ZnCl_2_ particles have the potential as safe and effective antimicrobials. Synthesis of these particles in conjunction with moist pig’s tissue illustrates these particles do not bind to moist tissue, making local CHX absorption less likely (Figure S7).

### 3.4. Release Kinetics of CHX Particles

**Figure 7:**
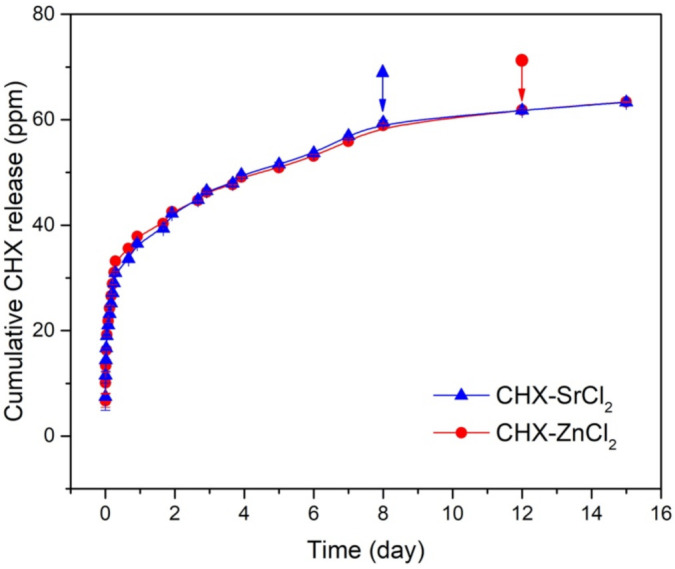
Cumulative CHX release curves for CHX-SrCl_2_ and CHX-ZnCl_2_ particles in PBS. Arrows with red round or blue triangle represent the last time point of the release CHX concentration higher than MIC.

The CHX-SrCl_2_ and CHX-ZnCl_2_ particles both showed a rapid CHX release in the first day, followed by a sustained release until the end of the assay as shown in Figure 7. The sustained CHX release behavior was associated with the unique interconnected structure of the novel CHX particles whose dissolution process initiates from the particle interior [70].

To inhibit bacterial growth, a sufficient dosage for an effective duration is required locally at the infection site to prevent secondary infections [70]. When the CHX concentration in the infection area is, however, too high, both tissue cells and bacteria can be eliminated [25]. CHX digluconate mouth rinses (0.12–0.2%) indicate a substantivity <12 h, requiring multiple daily applications to be effective [71]. The CHX-SrCl_2_ offered an effective sustained release up to 8 days, as the CHX concentration of released solution was below MIC (<2.5 ppm) after 8 days. The CHX-ZnCl_2_ showed a similar sustained release pattern (above MIC) up till day 12 (2.96 ppm). This may be particularly beneficial in prevention of recurrent infections and maintenance of oral hygiene. The novel particles offer an effective CHX release up to 8 days for CHX-SrCl_2_ and 12 days for CHX-ZnCl_2_ particles, which may be particularly beneficial in prevention of recurrent infections and maintenance of oral hygiene. Sustained release delivery systems also allow a better antibacterial efficacy against bacteria [25,72]. The calculated chlorhexidine content in the CHX-SrCl_2_ and CHX-ZnCl_2_ particles was around 98.6 wt% and 99.1 wt%, respectively, which was higher than many reported carriers [73]. Moreover, the CHX-SrCl_2_ or CHX-ZnCl_2_ particles were able to release additional antibacterial ions. CHX-SrCl_2_ particles contain strontium (Table S3) potentially available for release as an antibacterial agent, with the effects enhanced by the presence of fluoride [74]. Further work is however needed to assess any effects on bone regeneration at this Sr concentration present.

**Figure 8:**
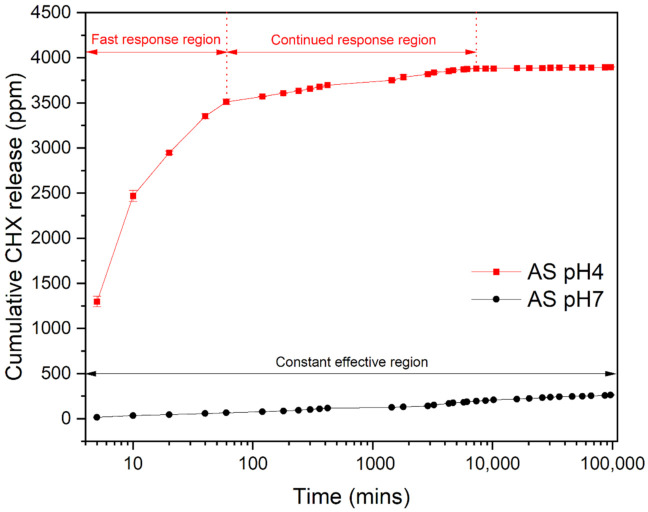
Cumulative zinc release from CHX-ZnCl_2_ particles in artificial saliva (pH = 4 or pH = 7).

The CHX-ZnCl_2_ particles had a higher CHX and Zn release rate in artificial saliva (pH 4), when compared to pH 7 (Figure 8 and Figure S8). After 7 h, the overall zinc released was 140 ppm for acidic artificial saliva (pH = 4), which was double that for neutral pH = 7 saliva (73 ppm). Figure 8 indicates a lower sustained CHX release pattern in a pH 7 environment, whilst for pH 4, a higher and responsive CHX release in the initial stages. However, after the initial CHX responsive release in AS (pH 4), an effective antibacterial concentration could be maintained for 5 days. After 40 days the cumulative CHX release reached 3888 ppm (AS = pH 7) versus 281 ppm for AS (pH 4).

The study of the release kinetics of the CHX particle in an acidic environment is of great importance, because caries or tissue inflammation are present in an acidic environment [75]. Specifically, during the caries and tissue inflammation process, there is a pH reduction due to activity of mutans streptococci and lactobacilli [76], which triggers the local acidification processes, encouraging acid-tolerant pathogenic bacteria to colonize [77]. The responsive and increased CHX release made available by the novel CHX-ZnCl_2_ particles is useful in this situation, particularly against drug resistant biofilms associated with surgical/recurrent infections [78]. In acidic condition (pH 4), the CHX can be rapidly released, providing an initial high dose of antibacterial drug, followed by a sustained and effective CHX release up to 5 days. This should be sufficient to arrest bacterial infections and maintain oral hygiene. In addition, if there were no potential infections (pH 7 environment) CHX would be released in a sustained manner for 19 days (at MBC level) maintaining oral hygiene, but available for smart release to eliminate any early-stage infections.

An acid environment may favor the dissolution of chlorhexidine [79], as it consists of two ionizable guanidine moieties [80] that demonstrate alkalinity [81]. The enhanced release rate of CHX will therefore also affect the zinc release behavior due to its dissolution. Furthermore, when AS (pH 7) contacted the surface of the hydrophilic CHX-ZnCl_2_ particles, zinc ions were released, due to the high surface area of the unique porous and interconnected particle structure. When the AS was acidic (pH 4), the release rate of zinc ions was accelerated, suggesting that coordination bonds in the CHX-ZnCl_2_ particle deteriorated more rapidly in the acidic environment [82], thus encouraging the release of Zn^2+^ from the lattice of the CHX-ZnCl_2_ particle. The drug release of the novel CHX-ZnCl_2_ particles can therefore vary with the surrounding pH values, enabling a responsive and controlled release of CHX and Zn. This is useful in the treatment of patients who are susceptible to bacterial infections, so early-stage disease effects are arrested or reversed. In particular, a reduction in oral bacterial load in COVID-19 patients may prevent pneumonia and acute respiratory distress syndrome, which can exacerbate patient morbidity [83]. It is noteworthy to mention that CHX-SrCl_2_ and CHX-ZnCl_2_ particles may have multiple applications including antibacterial mouth rinses, gels, cements, or coatings to take advantage of the sustained/smart release, or possible additional functions such as promoting bone proliferation [84]. The authors have incorporated novel CHX particles into injectable commercial gels for sustained drug delivery applications in a next step. This is expected to achieve long term sustained CHX release and antimicrobial efficacy, which is useful in the prevention of surgical and periodontal infections.

## 4. Conclusions

The current antibacterial CHX-SrCl_2_ and CHX-ZnCl_2_ formulations provide a cost effective and efficient drug synthesis route, whose size could be manipulated by adjusting synthesis time and temperature. They were effective against a range of oral pathogens with reduced cytotoxicity, extended duration, and with a smart pH responsive drug/ion release for the CHX-ZnCl_2_ particles. These particles show great potential for a new responsive treatment modality for the prevention or reversal of carries and infections in medicine and dentistry.

## Figures and Tables

**Table 1:** MIC and MBC of CHX-SrCl_2_, CHX-ZnCl_2_, and CHXD against *P. gingivalis* and *A. actinomycetemcomitans*.

Concentration of CHX-SrCl_2_, CHX-ZnCl_2_ or CHXD	24 h	48 h
0.00025%	MIC	MBC
0.0005%	MBC	MBC
0.001%	MBC	MBC

**Table 2:** MIC and MBC of CHX-ZnCl_2_, CHX-SrCl_2_, and CHXD against *F. nucleatum subsp. polymorphum (strain—ATCC10953)*.

Concentration of CHX-SrCl_2_, CHX-ZnCl_2_, or CHXD	24 h	48 h
0.0005%	MIC	MIC
0.001%	MBC	MBC
0.002%	MBC	MBC

## Data Availability

The data presented in this study are available on request from the corresponding author.

## Supplementary Materials

The following are available online at https://www.mdpi.com/article/10.3390/pharmaceutics13111799/s1, Figure S1: EDX analysis of the CHX-SrCl_2_ particles, Figure S2: EDX analysis of the CHX-ZnCl_2_ particles, Figure S3: Images for CHX-ZnCl_2_ particles at different reaction times, Figure S4: SEM images of CHX-SrCl_2_ particles, CHX-ZnCl_2_ particles at different synthesis temperatures, Figure S5: Antimicrobial assays on *Porphyromonas gingivalis*, *A. actinomycetemcomitans* and *F. nucleatum subsp. Polymorphum*, Figure S6: Effect of CHXD; CHX-SrCl_2_ and CHX-ZnCl_2_ particle on cellular viability. Figure S7: SEM images for treated pig tissue. Figure S8: Cumulative Zinc release curve for CHX-ZnCl_2_ particles in AS (pH = 7 or pH = 4). Table S1: UV-Vis schedule for the release assay, Table S2: EDS element analysis for the CHX-ZnCl_2_ particle, Table S3: EDS element analysis for the CHX-SrCl_2_ particle.
