# Supplementary material for: Synthesis, Drug Release, and Antibacterial Properties of Novel Dendritic CHX-SrCl2 and CHX-ZnCl2 Particles
Source: Pharmaceutics. 2021 Oct 27;13(11):1799. doi: 10.3390/pharmaceutics13111799 (PMC8625704; doi:10.3390/pharmaceutics13111799)
Supplement: Supplementary file 1 [file pharmaceutics-13-01799-s001.zip › pharmaceutics-1406106-supplementary.pdf]

pharmaceutics

Supplementary Materials: Synthesis, Drug Release and Antibacterial Properties of Novel Dendritic CHX-$SrCl_2$ and CHX-$ZnCl_2$ Particles

Rui Sun, Jiaxin Zhang, Robert A. Whiley, Gleb B. Sukhorukov and Michael J. Cattell

Table S1. UV-Vis schedule for the release assay.

Week	Day	Time interval (mins/hours)
1st Week	1st Day (Monday)	5 mins
		10 mins
		20 mins
		40 mins
		60 mins (1 hour)
		120 mins (2 hours)
		180 mins (3 hours)
		240 mins (4 hours)
		300 mins (5 hours)
		360 mins (6 hours)
		420 mins (7 hours)
	2nd Day (Tuesday)	2 readings (10:00 and 16:00)
	3rd Day (Wednesday)	2 readings (10:00 and 16:00)
	4th Day (Thursday)	2 readings (10:00 and 16:00)
	5th Day (Friday)	2 readings (10:00 and 16:00)
	6th Day (Saturday)	1 reading (12.00)
	7th Day (Sunday)	1 reading (12.00)
Week 2–4	Monday	1 reading (12.00)
	Friday	1 reading (12.00)

Table S2. EDS element analysis for the CHX-$ZnCl_2$ particle.

Element	Weight%	Atomic%
O K	48.39	69.03
Cl K	43.95	28.29
Zn K	7.66	2.68
Totals	100.00	

Table S3. EDS element analysis for the CHX-$SrCl_2$ particle.

Element	Weight%	Atomic%
O K	54.49	73.67
Cl K	41.56	25.36
Sr L	3.95	0.98
Totals	100.00	

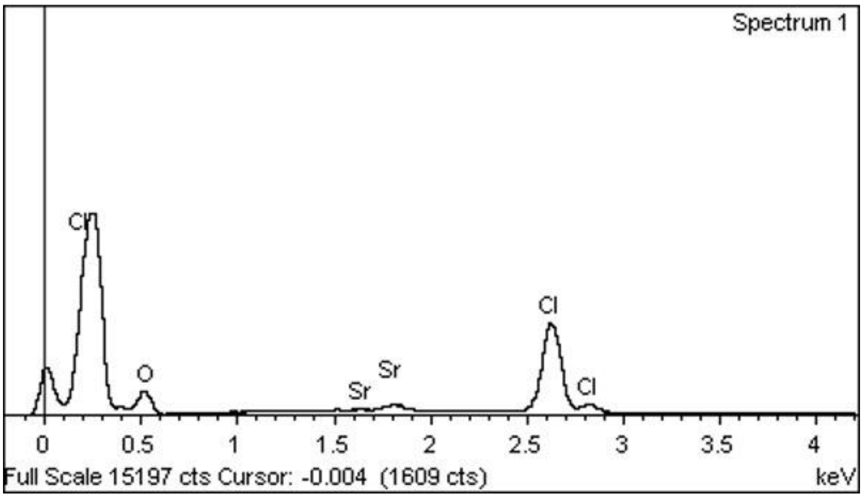

Figure S1. EDX analysis of the CHX-$SrCl_2$ particles.

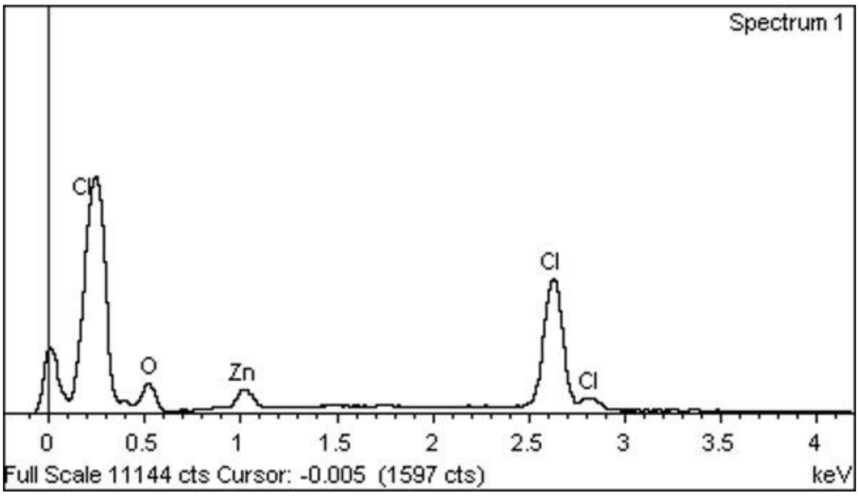

Figure S2. EDX analysis of the CHX-$ZnCl_2$ particles.

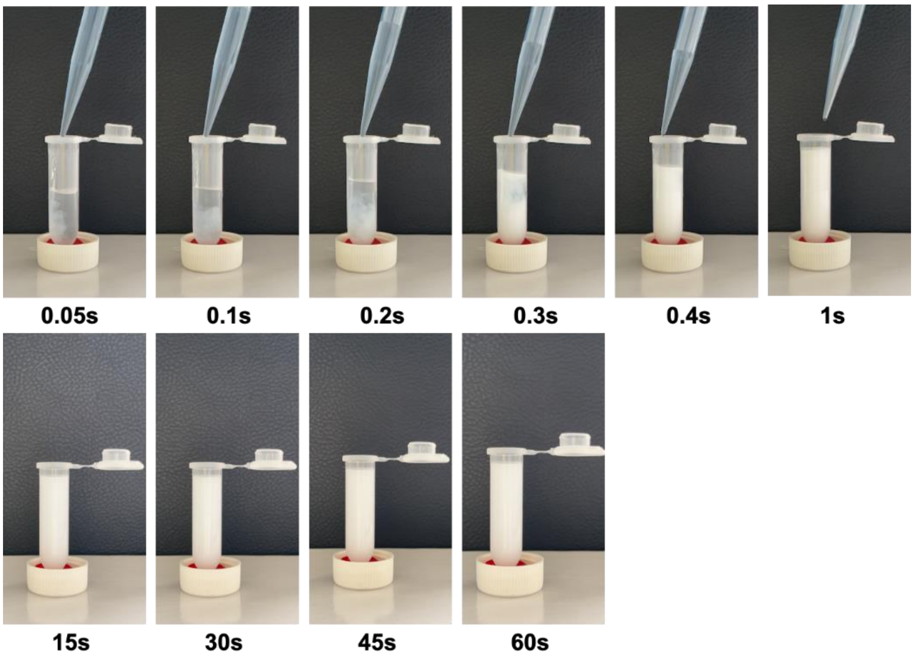

Figure S3. Images for CHX-$ZnCl_2$ particles at different reaction times.

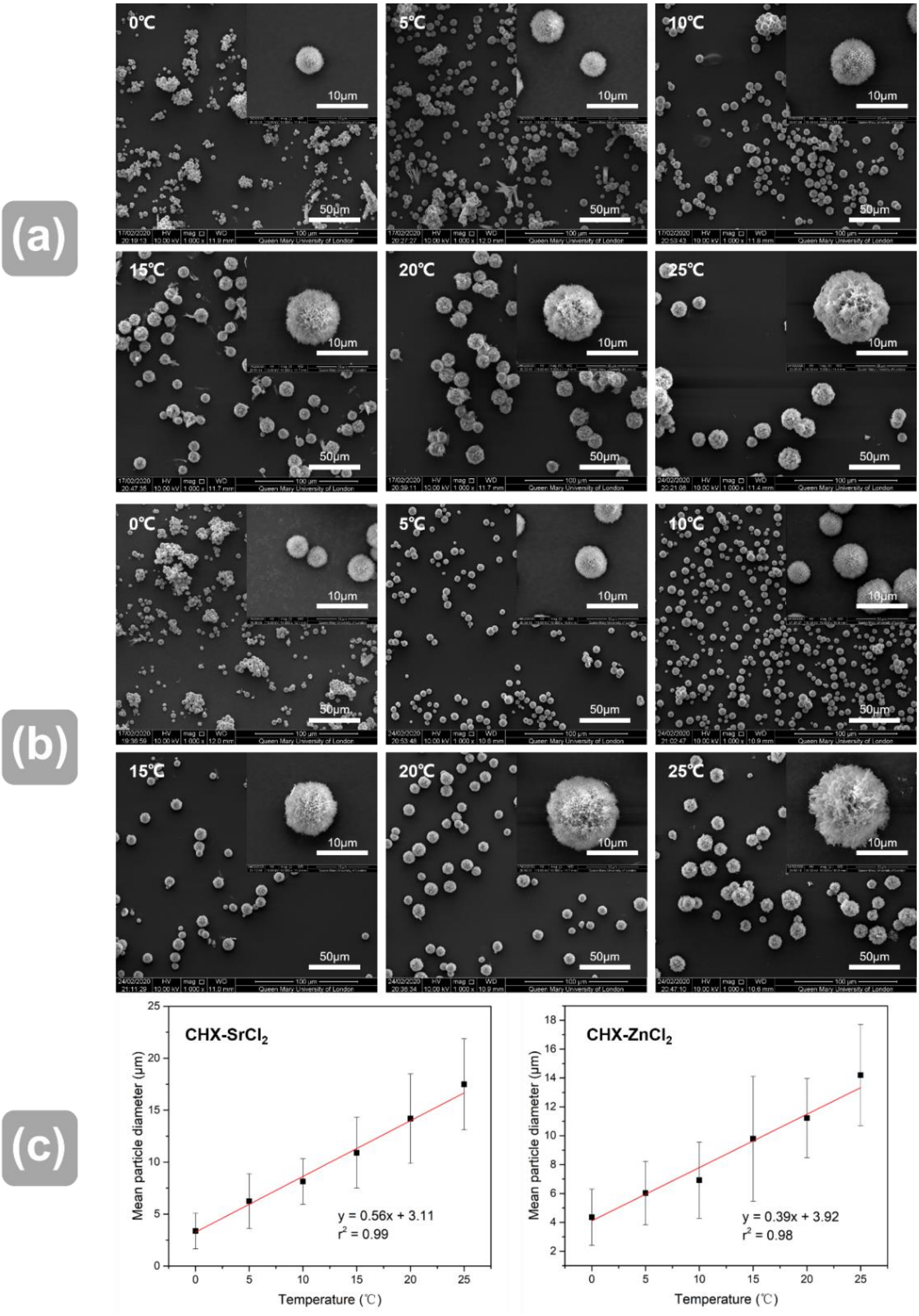

Figure S4. SEM images of: (**a**) CHX-$SrCl_2$ particles; (**b**) CHX-$ZnCl_2$ particles at different synthesis temperatures; (**c**) Plots showing the correlation between the Mean particle diameter and temperature for both CHX-$SrCl_2$ and CHX-$ZnCl_2$ particles.

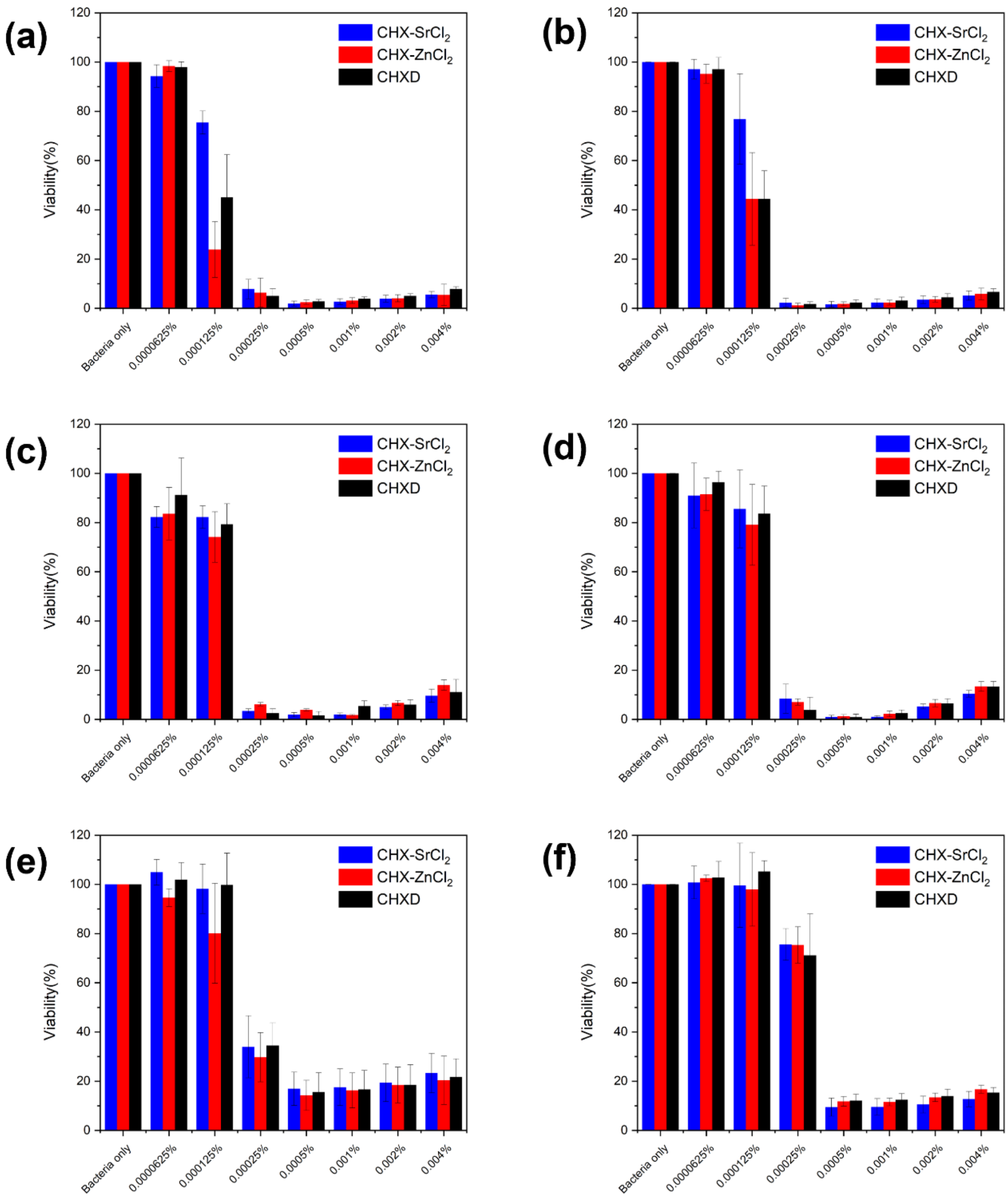

Figure S5. (**a**); Antimicrobial assays on *Porphyromonas gingivalis (strain- 381)* at 24 h (**b**); and 48 h (**c**); Antimicrobial assay on *A. actinomycetemcomitans (strain-Y4)* at 24 h and (**d**); 48 h (**e**); Antimicrobial assays on *F. nucleatum subsp. polymorphum (strain-ATCC)* at 24 h and (**f**); 48 h.

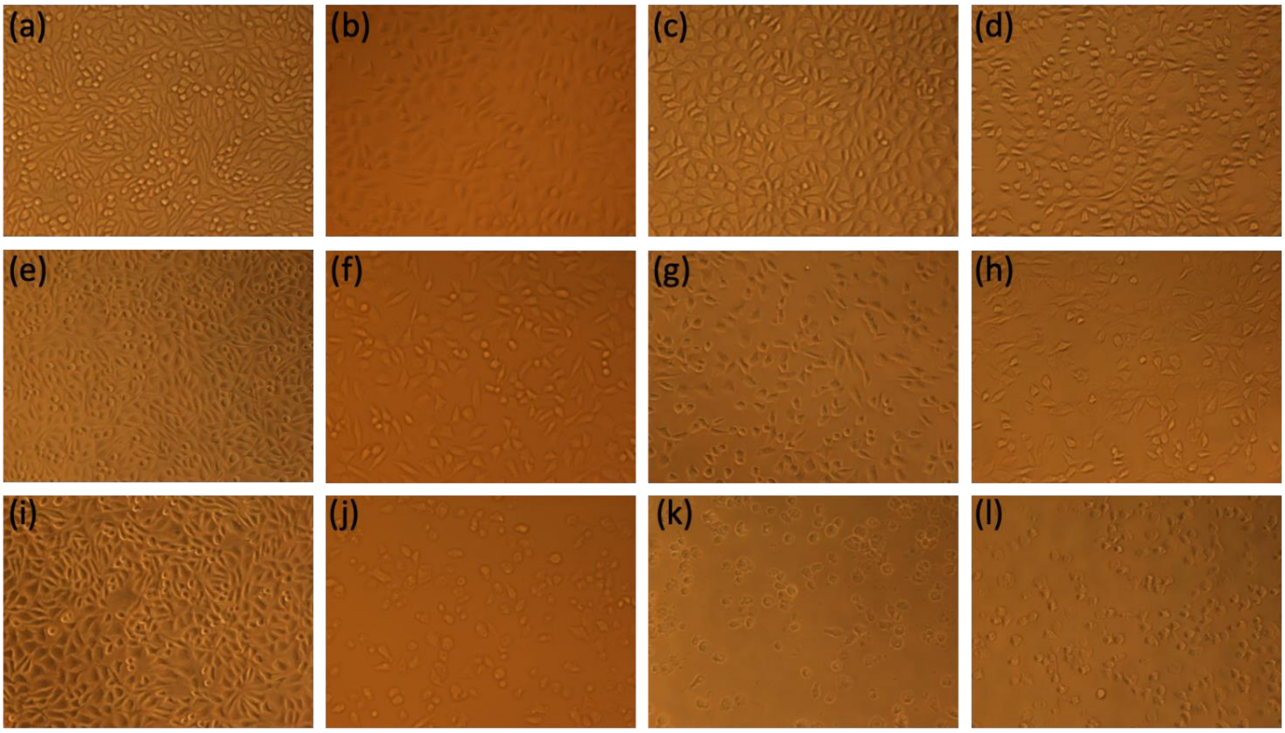

Figure S6. Effect of CHXD; CHX-$SrCl_2$ and CHX-$ZnCl_2$ particle on cellular viability. The Fibroblast like cell lines (L929) were treated with **a**, **e** and **i**) untreated, **b**) 0.00025% CHX-$SrCl_2$ particles, **c**) 0.00025% CHX-$ZnCl_2$ particles, **d**) 0.00025% CHXD particle for 48 hours, **f**) 0.0005% CHX-$SrCl_2$ particles, **g**) 0.0005% CHX-$ZnCl_2$ particles, **h**) 0.0005% CHXD particle for 48 hours. **j**) 0.001% CHX-$SrCl_2$ particles, **k**) 0.001% CHX-$ZnCl_2$ particles, **l**) 0.001% CHXD particle for 48 hours.

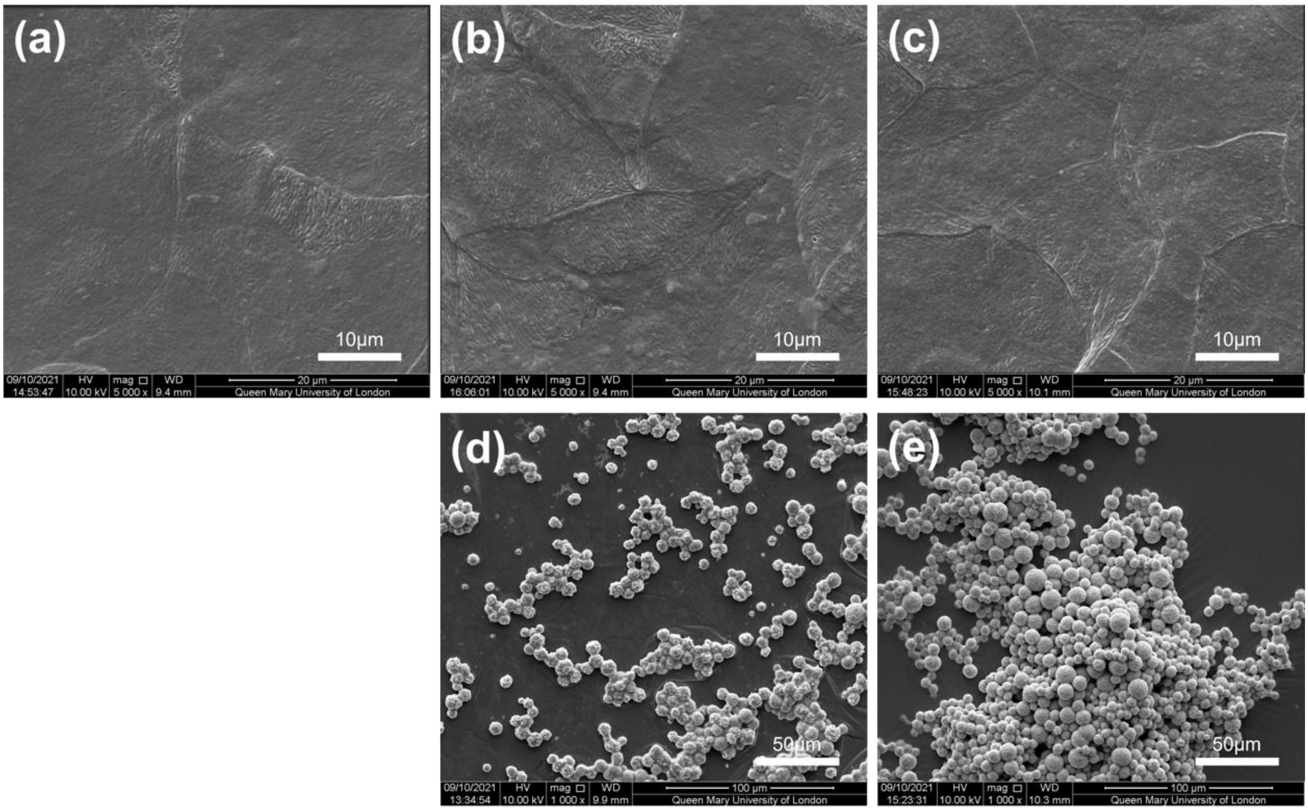

Figure S7. **a**) SEM images for pig's tissue without any coating, **b**) CHX-$SrCl_2$ particles coating on the moisturized pigs tissue after rinsing, **c**) CHX-$ZnCl_2$ particles coating on the moisturized pigs tissue after rinsing, **d**) CHX-$SrCl_2$ particles formed on separate SEM stub, e) CHX-$ZnCl_2$ particles formed on the on the separate SEM stub.

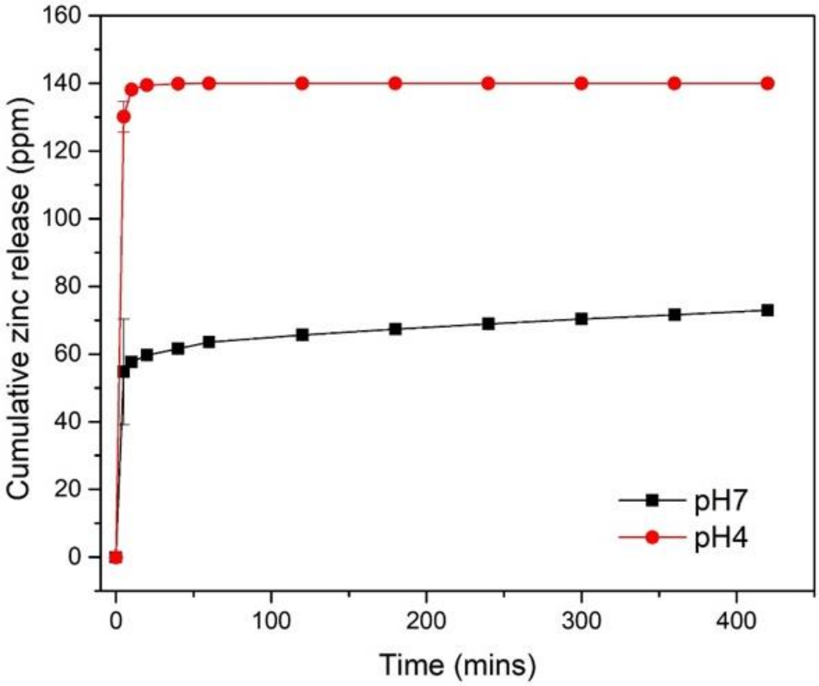

Figure S8. Cumulative Zinc release curve for CHX-$ZnCl_2$ particles in AS (pH=7 or pH=4).
